# A comparison of health-related quality of life (health utility) between insulin degludec and insulin glargine: a meta-analysis of phase 3 trials

**DOI:** 10.1111/dom.12086

**Published:** 2013-04-01

**Authors:** N Freemantle, M Evans, T Christensen, M L Wolden, J B Bjorner

**Affiliations:** 1 Department of Primary Care and Population Health, UCL Medical SchoolLondon, UK; 2 University Hospital LlandoughCardiff, UK; 3 Novo Nordisk A/SBagsværd, Denmark; 4 OptumLincoln, RI, USA; 5 Department of Public Health, University of CopenhagenCopenhagen, Denmark

**Keywords:** degludec, diabetes, glargine, health utility, insulin, quality of life, SF-36

## Abstract

**Aim:**

To evaluate health-related quality of life (health utility) scores in patients with diabetes receiving insulin degludec (IDeg) or insulin glargine (IGlar).

**Methods:**

Patient-level data from six, randomized, controlled, open-label, multicentre, confirmatory, treat-to-target trials of 26- or 52 weeks' duration were pooled in this analysis. The Short Form 36 (SF-36) version-2 health questionnaire was completed by patients at baseline and end-of-trial. SF-36 scores for 4001 individual patients were then mapped onto the EuroQol-5D health utility scale, which has a range from −0.59 (a state worse than death) to 1.00 (perfect health).

**Results:**

IDeg treatment exhibited a significant improvement in health status of 0.005 (CI: 0.0006; 0.009) points compared with IGlar (p < 0.024). Gender, region, trial and age also had a significant influence on estimated utility scores as did baseline utility scores, p < 0.05. Prior to the removal of interaction variables a difference of 0.008 points was observed, p < 0.045. Previous insulin treatment did not have an impact on the final outcome.

**Conclusion:**

This study shows that IDeg is associated with a modest, but statistically significant, improvement in health utility compared with IGlar in patients with diabetes.

## Introduction

Diabetes places a significant burden upon health-related quality of life (HRQoL), with patients experiencing a reduction in both total and healthy life years as a result of disability and related co-morbidities [1]. Additional challenges arise during the management of diabetes, where the fear of hypoglycaemia, fear of injections and complex treatment regimens are major concerns among patients [2–4]. These factors have a negative impact on HRQoL, and incur economic costs to the individual and the healthcare system [5–8]. Increasingly, the health economic value attached to health status is used to inform the decisions of healthcare payers when assessing the cost-effectiveness of new treatments [9]. For the purpose of health economics, HRQoL may be expressed as a single preference value, health utility, where zero is equivalent to death and one represents perfect health. Typical values for diabetes patients without complications range between 0.9 (type 1) and 0.85 (type 2), and are lower in those with complications [10,11]. Quality-adjusted life years (QALYs), a widely used measure of health improvement, are formed by combining health utility scores with a relevant time horizon [12].

Insulin degludec (IDeg) is a new-generation ultra-long acting basal insulin that forms soluble multi-hexamers upon subcutaneous injection, achieving a stable glucose-lowering effect lasting beyond 42 hours [13,14]. The safety and efficacy of IDeg has been compared with insulin glargine (IGlar) in clinical trials, where IDeg has shown lower rates of day-to-day and hour-to-hour blood-glucose variability [15], and a reduced risk of hypoglycaemia at equivalent levels of glycaemic control [16–20]. Furthermore, IDeg has previously been shown to improve QoL compared with IGlar in both type 1 diabetes mellitus (T1D) and type 2 diabetes mellitus (T2D) [17,19,21,22]. The aim of this meta-analysis was to evaluate health utility scores in patients with diabetes receiving IDeg or IGlar.

## Methods

### Study Population and Clinical Endpoints

The IDeg clinical trial programme used similar methodologies across trials to allow data to be grouped for meta-analyses. All phase 3a clinical trials where IDeg once daily (OD) was compared with IGlar OD, and where QoL was evaluated, were included in this analysis (Table 1) [17,18,23–25]. Patient-level data (n = 4001) from six, randomized, controlled, open-label, multicentre, confirmatory, treat-to-target trials of 26- or 52 weeks' duration were pooled in this analysis. These included one trial in T1D using basal−bolus (BB) therapy (id #3583 [18]), one in T2D using BB (id #3582 [17]), and four in T2D using basal insulin supported oral therapy (id #3579, 3586, 3668, 3672 [19,23–25]). Patients received either IDeg or IGlar OD in each of these trials. Basal insulin doses were administered using either the FlexTouch® (Novo Nordisk, Bagsvaerd, Denmark) or SoloStar® (sanofi-aventis, Paris, France) pen injectors for IDeg and IGlar, respectively – vials/syringes were not used in any of the studies. Inclusion criteria required patients to be ≥18 years of age, with a duration of diabetes ≥6 months, glycated haemoglobin (HbA1c) ≤11% and a body mass index (BMI) ≤45 kg/m^2^. In study 3668 [24], there were three treatment arms: IDeg fixed dosing, IGlar fixed dosing and IDeg flexible dosing. In the fixed-dosing arms, as with all other trials, the basal insulin dose was administered at the same time every day. In the IDeg flexible dosing arm, insulin doses were administered at alternating 8- and 40-h intervals. To prevent differences in the timing of insulin dose administration from confounding the results, the IDeg flexible dosing arm in study 3668 was excluded from this meta-analysis. A seventh phase 3a study (id #3770 [26]) was not included in this meta-analysis because QoL data were not recorded. This analysis included both insulin-experienced and insulin-naïve patients. Individuals were excluded from the trials if they had recurrent severe hypoglycaemia (≥1 event, requiring third-party assistance, in the preceding 12 months). Basal insulin doses were titrated to a target fasting plasma glucose (FPG) concentration of 90 mg/dl (5 mmol/l). Glycaemic control was evaluated via measurement of HbA1c and FPG concentrations. Safety analysis included the recording of confirmed hypoglycaemic events. These were defined as blood glucose concentrations <56 mg/dl (3.1 mmol/l) or requiring third-party assistance, and were considered as nocturnal if onset occurred between 00:01 and 05:59 (inclusive) hours. Owing to the global nature of the phase 3a programme, health questionnaires were translated and validated in the appropriate languages. Detailed descriptions of the study methodology are available in previously published literature [17–19].

**Table 1:** Baseline characteristics for all patients in each of the six trials included in the meta-analysis

Study id	Short trial name	Prestudy treatment numbers	Age (years)	Diabetes duration (years)	BMI (kg/m^2^)	HbA1c (%)	FPG (mg/dl)
3582 [17]	BEGIN™ T2 BB	992	58.9 ± 9.3	13.5 ± 7.3	32.2 ± 4.6	8.3 ± 0.8	166 ± 56
3583 [18]	BEGIN™ T1 BB LONG	629	43.0 ± 13.6	18.9 ± 12.0	26.3 ± 3.8	7.7 ± 1.0	168 ± 74
3579 [19]	BEGIN™ Once Long	1030	59.1 ± 9.8	9.2 ± 6.2	31.1 ± 4.7	8.2 ± 0.8	175 ± 47
3586 [23]	BEGIN™ ONCE ASIA	435	58.6 ± 9.9	11.6 ± 6.5	25.0 ± 3.6	8.5 ± 0.8	153 ± 36
3668 [24]	IDeg Flexible versus IDeg Fixed and IGlar in T2D	458	56.4 ± 9.6	10.6 ± 6.7	29.6 ± 4.7	8.4 ± 0.9	160 ± 49
3672 [25]	BEGIN™ LOW VOLUME	457	57.5 ± 9.2	8.2 ± 6.2	32.4 ± 5.4	8.3 ± 0.9	173 ± 49
Total	—	4001	56.0 ± 11.7	12.1 ± 8.4	30.0 ± 5.3	8.2 ± 0.9	166.9 ± 54.1

Data are mean values ± standard deviation unless otherwise stated.

BB, basal–bolus; BMI, body mass index; FPG, fasting plasma glucose; IDeg, insulin degludec; IGlar, insulin glargine; n/a, data not available; T2D, type 2 diabetes mellitus.

In all trials, IDeg achieved non-inferiority of glycaemic control, as measured by change in HbA1c from baseline compared with IGlar – as would be expected in treat-to-target studies. FPG and nocturnal hypoglycaemic events were numerically or significantly reduced, and overall confirmed hypoglycaemic events were equal to or fewer for IDeg compared with IGlar. Detailed results of individual trials have been published [17–19].

### Health Utility (HRQoL) Assessment

**Figure 1:**
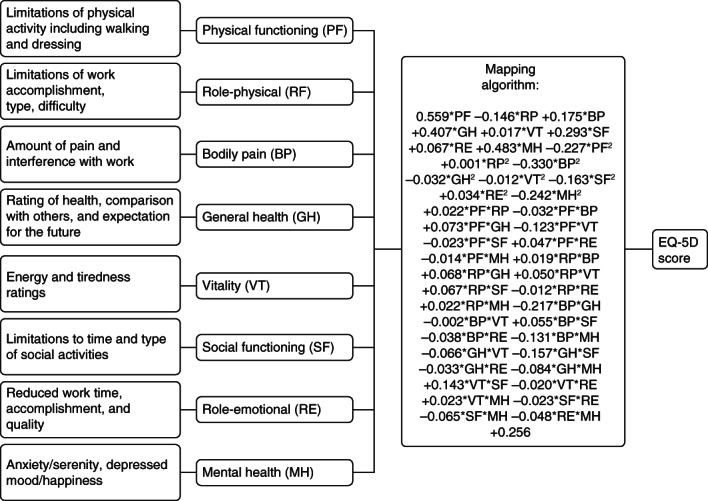
Short Form 36 (SF-36) to EuroQol-5D (EQ-5D) conversion algorithm: integrated with outline of SF-36 questionnaire. To generate the EQ-5D, each SF-36 scale is rescaled onto a zero (worst health) to one (best health) scale before applying the mapping algorithm.

The Short Form 36 (SF-36) version-2 health questionnaire was completed by patients at baseline and end-of-trial. The SF-36 comprises 36 questions, which yield scores for eight scales and which are summarized into two health measures concerning physical and mental health [27]. As with health utility, an increase in SF-36 scores represents an improvement in health; however, SF-36 scores are not based on individual preferences. In this analysis, generic HRQoL scores from the SF-36 instrument were converted into EuroQol-5D, (EQ-5D) health utility scores [28], by use of a method adopted from Rowen et al. [29] (figure 1). To generate the EQ-5D, each SF-36 scale is rescaled onto a −0.59 (worst health) to 1 (best health) scale before applying the mapping algorithm. Rowen et al. examined a number of different models for mapping SF-36 scores on to EQ-5D, and compared the performance of the various specifications via their ability to accurately predict EQ-5D scores from SF-36 scores using a ‘real-world’ dataset. Their preferred model (based on observed vs. predicted precision) is a random effect, generalized least squares model, where SF-36 domain scores, squared domain scores and interactions between domain scores are included [29]. It was not appropriate to use an ordinal least squares model, due to a lack of normality across the disease severity range and interaction effects between dimensions. The preferred prediction model had the highest predictive value in less severe health states compared with more severe health states. Health status scores in the present, IDeg, clinical trials match the range where predictive value is greatest, therefore this model was deemed appropriate. The National Institute for Health and Clinical Excellence (NICE) recommends the use of EQ-5D when measuring health utility. When EQ-5D has not been directly assessed, NICE favours the adoption of mapping scores to EQ-5D over the use of alternate measures, such as SF-6D [30]. Patient-level SF-36 data from the individual trials were then mapped to EQ-5D. This enabled the direct estimation of a utility value for individuals (EQ-5D individual mapping), and permitted conditioning of relevant patient characteristics on an individual subject-level detail in the final statistical model.

Two alternative utility models were applied to test the sensitivity of our approach, but were also relevant in assessing the robustness of the parameter estimate derived from the primary mapping model. The SF-6D is a widely used direct index (e.g. not mapped via EQ-5D) which as with the EQ-5D is also based on respondent preferences [31]. The index comprises six dimensions and can yield 18 000 unique health states/utility values. Lastly, an algorithm mapping mean SF-36 scores to the EQ-5D (EQ-5D aggregate mapping) was also tested as alternative to the primary model [32].

### Statistical Analysis

**Figure 2:**
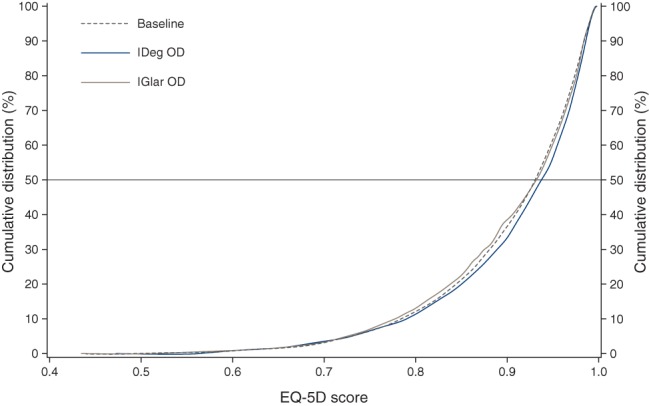
EuroQol-5D (EQ-5D) scores − empirical distribution function.

Data from individual trials were pooled using a fixed-effects model. SF-36 scores for 4001 individual patients were mapped onto EQ-5D and a generalized linear regression was applied subsequently, using SAS v9.1.3 (SAS Institute, Cary, North Carolina, USA), to estimate the treatment effect of IDeg and IGlar in terms of utility. EQ-5D scores can exhibit a ceiling effect when a large proportion of subjects describe perfect health (=1). However, ceiling effects did not seem to be an issue in this study population. Although a large proportion of patients has scores at the high end (the percentage of patients having values >0.95 was 63.5 and 65.9% at baseline, and 58.0 and 61.1% at follow-up, for IDeg and IGlar, respectively) no patients reached the ceiling at baseline or follow-up in either treatment group (figure 2). The absence of patients reaching the ceiling is a result of the EQ-5D values being modelled from the SF-36 scale. Furthermore, none of the patients reached the ceiling on the SF-36 scale; therefore, it is unlikely that this would be the case for the EQ-5D predicted values. It should, however, be noted that a fairly large proportion are at the higher end of the scoring range as would be expected in patients with diabetes who do not have major health complications [33,34]. We conducted an analysis of covariance, using end-of-trial utilities as endpoint and baseline utility, treatment (IDeg vs. IGlar), trial, region, gender, age and previous insulin treatment as independent variables. Also, the initial model included a vector of interaction variables of treatment by trial. These variables were used to examine whether treatment results differed by trial.

As a sensitivity analysis, a linear model with interaction variables of key clinical markers was specified to test for statistical prediction power of the utility difference. Age is normally a pivotal driver of utility; therefore, several different specifications of age (log, exponential or quadratic) were assessed as alternatives to linear age in the model.

SF-6D utility scores were calculated using the scoring algorithms developed by Brazier et al. [31]. The change in score was analysed using the same model as was applied for EQ-5D. Finally, we derived EQ-5D scores through aggregate mapping, by multiplying the raw non-normalized SF-36 scores onto the parameter estimates provided in Ara et al. [32].

## Results

### Baseline Characteristics

The study population consisted of 4001 patients across six trials. For the combined study population, the mean (±s.d.) age was 56.0 ± 11.7 years, duration of diabetes 12.1 ± 8.4 years, HbA1c of 8.2 ± 0.9 mmol/mol, FPG 166.9 ± 54.1 mg/dl and BMI of 30.0 ± 5.3 kg/m^2^. Baseline values for the individual trials are shown in Table 1. EQ-5D individual mapping scores at baseline and follow-up are shown in Table 2.

**Table 2:** Utility scores at baseline and follow-up for the EQ-5D individual mapping algorithm

	IDeg		IGlar	
Study id	Baseline	End-of-trial	Baseline	End-of-trial
3582 [17]	0.8843 ± 0.0931	0.8837 ± 0.0977	0.8858 ± 0.0948	0.8784 ± 0.0933
3583 [18]	0.9436 ± 0.0636	0.9430 ± 0.0599	0.9414 ± 0.0607	0.9342 ± 0.0711
3579 [19]	0.8958 ± 0.0867	0.9009 ± 0.0894	0.8951 ± 0.0912	0.8933 ± 0.0945
3586 [23]	0.9208 ± 0.0575	0.9286 ± 0.0618	0.9211 ± 0.0632	0.9273 ± 0.0566
3668 [24]	0.8958 ± 0.0797	0.9030 ± 0.779	0.8921 ± 0.0810	0.8972 ± 0.0912
3672 [25]	0.8957 ± 0.0887	0.9080 ± 0.0810	0.8889 ± 0.0858	0.8963 ± 0.0912

Values are mean ± standard deviation.

IDeg, insulin degludec; IGlar, insulin glargine.

### Health Utility (EQ-5D)

In the sensitivity analysis, none of the alternative age specifications markedly improved the model's goodness-of-fit (assessed by the Akaike Information Criterion (AIC) score, [35]). Consequently, linear age was retained in the final model. Testing for interaction variables did not yield a significant effect, therefore the final model proceeds with the assumption that there is no difference in effect between the treatment settings.

Cumulative treatment scores for the predicted EQ-5D are shown in figure 2. IDeg treatment exhibited a significant improvement in health status of 0.005 (CI: 0.0006; 0.009) points compared with IGlar (p < 0.024) (Table 3). Gender, region, trial and age also had a significant influence on estimated utility scores as did baseline utility scores, p < 0.05 (Table 3). Prior to the removal of interaction variables a difference of 0.008 points was observed, p < 0.045 (Table 3). Previous insulin treatment (Yes/No) did not have an impact on the final outcome (Table 3).

**Table 3:** Overview of parameter estimates included in QALY regression analysis before and after backward elimination of candidate explanatory variables

Variable	Parameter	Estimate (s.e.) before reduction	p value	Estimate (s.e.) in final model	p value
Intercept	—	0.313 (0.016)	—	0.315 (0.016)	—
Treatment	—	—	0.045	—	0.024
	IDeg	0.008 (0.006)	—	0.005 (0.002)	—
	IGlar	0	—	0	—
Gender	—	—	0.003	—	0.003
	Female	−0.006 (0.002)	—	−0.006 (0.002)	—
	Male	0	—	0	—
Region	—	—	0.016	—	0.017
	Asia	−0.024 (0.010)	—	−0.023 (0.010)	—
	Europe	−0.015 (0.009)	—	−0.015 (0.009)	—
	Japan	−0.024 (0.012)	—	−0.024 (0.012)	—
	North America	−0.021 (0.009)	—	−0.021 (0.009)	—
	South Africa	−0.014 (0.010)	—	−0.013 (0.010)	—
	South America	0	—	0	—
Insulin naïve	—	—	0.329	—	—
	No	−0.006 (0.006)	—	—	—
	Yes	0	—	—	—
Trial	—	—	0.006	—	<0.0001
	T2D BOT (3579)	−0.007 (0.006)	—	−0.007 (0.003)	—
	T2D BB (3582)	−0.009 (0.008)	—	−0.015 (0.003)	—
	T1D BB (3583)	0.001 (0.009)	—	−0.005 (0.004)	—
	T2D BOT Asia (3586)	0.014 (0.009)	—	0.010 (0.008)	—
	T2D BOT Flex (3668)	0.002 (0.007)	—	−0.004 (0.005)	—
	T2D BOT U200 (3672)	0	—	0	—
Treatment by trial interaction	—	—	0.875	—	—
	IDeg × T2D BOT	−0.002 (0.007)	—	—	—
	IDeg × T2D BB	−0.001 (0.007)	—	—	—
	IDeg × T1D BB	−0.001 (0.008)	—	—	—
	IDeg × T2D BOT Asia	−0.006 (0.008)	—	—	—
	IDeg × T2D BOT Flex	−0.009 (0.008)	—	—	—
	IDeg × T2D BOT U200	0	—	—	—
	IGlar × T2D BOT	0	—	—	—
	IGlar × T2D BB	0	—	—	—
	IGlar × T1D BB	0	—	—	—
	IGlar × T2D BOT Asia	0	—	—	—
	IGlar × T2D BOT Flex	0	—	—	—
	IGlar × T2D BOT U200	0	—	—	—
Age	—	−0.0005 (0.0001)	<0.0001	−0.0005 (0.0001)	<0.0001
Baseline utility	—	0.712 (0.012)	<0.0001	0.712 (0.012)	<0.0001

p values are based on type III sums of squares test.

BB, basal–bolus; BOT, basal supported oral therapy; IDeg, insulin degludec; IGlar, insulin glargine; QALY, quality-adjusted life years; s.e., standard error; T1D, type 1 diabetes mellitus; T2D, type 2 diabetes mellitus.

The results of the alternative algorithms generally agreed with the findings of the primary EQ-5D mapping approach. Using the SF-6D and EQ-5D aggregate mapping, the improvement in health utility was 0.005 and 0.012, respectively. Although statistical analysis comparing the aggregate scores was not possible, the similarity between the three sets of results supports the validity of the mapping algorithm in this study (Table 4). A breakdown of the scores by treatment group for each algorithm is shown in Table 4.

**Table 4:** Sensitivity analysis for the EQ-5D individual mapping, EQ-5D aggregate mapping and SF-6D algorithms

		QALYs		
QALY gain calculation	Treatment regimen	EQ-5D individual mapping	EQ-5D aggregate mapping	SF-6D
Separate	T1D BB	0.007	0.007	0.005
	T2D BOT	0.004	0.015	0.005
	T2D BB	0.006	0.014	0.002
Aggregate	ALL	0.005*	0.012	0.005

BB, basal–bolus; BOT, basal supported oral therapy; EQ-5D, EuroQol-5D; QALY, quality-adjusted life years; SF-36, Short Form 36; T1D, type 1 diabetes mellitus; T2D, type 2 diabetes mellitus.

* Utility estimate used in the base case analysis.

## Conclusions

This study shows that IDeg is associated with a modest, but statistically significant, improvement in health utility compared with IGlar in patients with diabetes.

These results concur with those of recently published trials, where IDeg has shown improvements in HRQoL compared with IGlar [17,19,21,22], and go a step further by translating this into health utility scores. The clinical trials included in our meta-analysis are some of the first involving an injectable insulin to measure HRQoL, and advance the use of patient-reported outcomes (PRO) in evaluating the value of therapeutic innovations in patients with diabetes.

It has been suggested that the reduced rate of hypoglycaemia observed with basal insulin analogues versus human insulins may be linked to improvements in HRQoL [21,22,36,37]. Conversely, a Cochrane review of studies involving basal insulin analogues confirmed a reduction in the rate of hypoglycaemic events compared with neutral protamine Hagedorn insulin, but did not show a benefit to QoL as these trials had not incorporated PRO assessments [38]. This highlights the importance of considering HRQoL when designing and implementing studies assessing the clinical value of novel insulin preparations. As previously stated, hypoglycaemia and fear of hypoglycaemia, are both major contributors to reduced HRQoL in patients with diabetes; therefore, it is possible that the improvement in health utility for IDeg is due to a reduced rate of nocturnal hypoglycaemia. However, other factors not recorded in the clinical trials may have contributed to the documented difference. Although non-severe hypoglycaemic episodes (NSHEs) have a negative impact on HRQoL they are often under-reported due to the difficulty in defining/classifying events. This is an important consideration for future studies because any difference in the rate of NSHEs may influence health utility scores and productivity [7,39].

Significant improvements in overall physical health and reductions in body pain among patients with T2D [17,19,21] receiving IDeg made substantial contributions to the between-treatment difference in utility scores. These benefits may have been derived from differences in the preparation of the two insulins. Notably, localized injection-site pain has been reported among patients receiving IGlar [40].

For reasons of consistency, the flexible dosing potential of IDeg has not been explored in this meta-analysis, but it may offer further benefits to health utility through increased adherence and reduced anxiety over injection schedules. A separate study is warranted to investigate whether flexible dosing of basal insulin improves health utility compared with fixed-time dosing.

A central assumption in meta-analyses such as the present one is that a general treatment effect applies across the different populations and conditions of the individual studies. We did indeed find significant differences in baseline utility levels between regions and between trials. However, we controlled for these differences in the analyses and we did not find any indications of a differential treatment effect by trial.

This analysis provides an estimated value for the difference in health utility between IDeg and IGlar. Though modest, the estimated difference between treatments appears real. When preference-based measures are used in the process of allocating healthcare resources, it is the difference in cost-effectiveness (incremental cost per QALY) that is important, rather than the change in QoL [41]. It should also be noted that the reduced rate of hypoglycaemia in IDeg may provide further economic advantages through decreased cost of testing strips, improved productivity and a reduction in the number of admissions to emergency departments [7,42].

There were some limitations to this analysis, specifically the open-label design of the trials, which is often perceived as being vulnerable to bias. With this in mind, the SF-36 questionnaire was completed first at baseline, prior to randomisation, to limit brand-specific bias. In addition, the duration of the trials was sufficiently long that any emotions relating to starting or changing insulin regimens should have diminished by the end of the trial period. The fact that trial populations are subject to exclusion criteria, such as severe recurrent hypoglycaemia and diabetes-related complications, suggests that the patients included in this meta-analysis are likely to have higher baseline health utility scores than the mean for the general population of patients with diabetes. ‘Real-world’ evidence is necessary to confirm the results of these clinical trials.

The EQ-5D has attracted criticism for lacking a dimension for energy/vitality [43]. Given that vitality was significantly improved in IDeg SF-36 scores, but not captured by the conversion algorithm, the differences in utility scores reported here may be underestimated. Strengths of this study include the randomized controlled trial context, the preplanned nature of the analysis and the use of individual patient-level data.

In summary, the results of this preplanned meta-analysis show that IDeg treatment improves HRQoL compared with IGlar, at equivalent levels of glycaemic control. Future studies should seek to investigate any potential relationship between reduced rates of hypoglycaemia in patients treated with IDeg, and improvements in HRQoL.
